# Characterisation of Electro-Brush Plated Nickel Coatings on P-Type (Zr,Ti)Co(Sn,Sb) Half-Heusler Thermoelectric Materials for Stable Contact Layers

**DOI:** 10.3390/ma18225108

**Published:** 2025-11-10

**Authors:** Mikdat Gurtaran, Zhenxue Zhang, Xiaoying Li, Hanshan Dong

**Affiliations:** 1 Faculty of Engineering, Material Science and Engineering, Izmir Institute of Technology, 35433 İzmir, Türkiye; 2 School of Metallurgy and Materials, The University of Birmingham, Birmingham B15 2TT, UK; x.li.1@bham.ac.uk (X.L.);

**Keywords:** thermoelectric materials, half-Heusler, interfacial design, Ni coating, electro-brush plating

## Abstract

In this study, a highly conductive nickel (Ni) layer was deposited onto a P-type (Zr,Ti)Co(Sn,Sb) half-Heusler (HH) thermoelectric (TE) material using a low-cost electro-brush plating technique. Before depositing Ni on the TE material, the plating process was optimised on a stainless steel (SS) substrate. An optimal medium-rate deposition voltage of 6V was identified on the SS substrate, with the desired thickness, superior mechanical performance, reduced sheet resistance and surface roughness, and enhanced electrical conductivity. The optimised deposition condition was then applied to the P-type (Zr,Ti)Co(Sn,Sb) material, resulting in a Ni layer that significantly enhanced its electrical and thermal stability. After thermal exposure at 500 °C for 10 h, the Ni coating effectively protected the TE surface against oxidation and sublimation, suggesting that the interfacial contact properties of P-type (Zr,Ti)Co(Sn,Sb) TE material can be effectively enhanced by depositing a highly conductive, oxidation-resistant Ni layer using the cost-effective, straightforward electro-brush plating technique.

## 1. Introduction

Given the negative environmental impacts of widely used fossil fuels, it has become essential to develop technologies that rely on clean, renewable, and sustainable energy sources [1,2]. Among these alternatives, thermoelectric (TE) technology has gained considerable momentum over the past two decades [3,4]. This technology enables the conversion of waste heat into usable electrical energy, thereby increasing energy utilisation. However, the heat-to-electricity conversion efficiency of TE modules remains a challenge. In recent years, many researchers have focused on increasing the figure of merit (zT = S^2^σκ^−1^ T) value of TE materials [5,6,7,8], which is a key approach to improving module efficiency. Although zT—a function of the Seebeck coefficient (S), electrical (σ) and thermal conductivities (κ), and the absolute temperature (T)—has been significantly enhanced across a range of temperatures for various TE materials [9,10,11,12], improvements in practical heat-to-electricity conversion efficiency can be achieved through high-quality TE module design [13,14,15]. In addition to developing TE materials with high zT values, selecting suitable electrode materials and optimising contact layers with high electrical and thermal conductivity are also critical factors for enhancing TE module performance [16,17,18]. In this regard, Heusler and half-Heusler intermetallic compounds have attracted significant interest for energy conversion applications due to their crystal structures, which determine their electronic properties and provide an improved figure of merit at elevated temperatures [19,20]. Full Heusler alloys have the general composition X_2_YZ and adopt the L2_1_ structure, while half-Heusler alloys (general formula XYZ) crystallise in the corresponding C1*b* structure with a regular vacancy in one of the sublattices [21]. These compounds, based on transition metals (X,Y) and main-group elements (Z), are optimised for high Seebeck coefficients and electrical conductivity by controlling the band structure, carrier concentration, and phonon scattering [22]. In particular, half-Heusler compounds have gained increasing importance in thermoelectric research in recent years due to their high electrical conductivity and mechanical performance [23,24].

The contact between thermoelectric materials and electrodes is crucial for ensuring TE module integrity and enabling efficient electron transport through the TE material. The most common ways for bonding TE materials to electrodes are welding or soldering [25]. However, the contact interface may degrade under high-temperature static or cyclic oxidation, leading to reduced electrical and thermal conductivity. A widely adopted solution to this issue is the deposition of metallising coatings on TE materials. These coatings serve as contact layers at the TE-electrode interface, enhancing electrical and thermal conductivity while simultaneously protecting the TE material against oxidation. When designing contact layers, the operating temperature of the TE module must be carefully considered [26]. Single-layer metal contacts are generally sufficient for low-temperature applications, whereas multilayer metal contacts are preferred for high-temperature operations [27,28]. Such contact layers not only prevent chemical reaction between the electrode and high-entropy TE materials, but also preserve the chemical stability of the TE material, and ensure efficient heat and charge transport by maintaining low contact resistance [25]. Recently, nickel (Ni) [29,30], copper (Cu) [31] and cobalt (Co) [32] contact layers have emerged as the most promising contacting materials due to their high conductivities and strong adhesion to the TE substrates (see Table 1).

Recent studies have primarily employed conventional metallisation techniques, such as magnetron sputtering and thermal evaporation, to deposit metallic contact layers on TE materials [33,34]. In addition to the metallisation coatings, some studies directly contact the metallic foils to the substrate via mechanical bonding [35,36]. While these methods can produce uniform coatings, they often require high-vacuum systems, involve high processing costs, or induce thermal and mechanical stresses that can degrade the TE substrate (Table 1).

**Table 1:** Comparison of reported Ni metallisation methods and contact performance on thermoelectric materials.

Coating Material	Substrate Material	Production Method	Temperature (°C)	Coating Thickness (µm)	Advancements / Limitations	Ref.
Ti	Bi_2_Te_3_-based	Magnetron Sputtering	250	0.015	High thermal stability, low contact resistance/high working temperature, high cost	[37]
Ni/Co	Bi_2_Te_3_-based	Chemical	98	8–12	Uniform coating/requires deposition of the sublayer via sputtering	[29]
Ni/Cu	Mg_2_Si-based	Magnetron Sputtering	RT	0.2	Good adhesion, low contact resistance/high cost	[38]
Ni	Bi_2_Te_3_-based	Electro- chemical	90	0.3	Good adhesion strength, sufficient electrical properties/high working temperature, high cost	[39]
Ni	Bi_2_Te_3_-based	Arc-spraying	300 (ageing)	50	Good bonding strength, high thermal resistance/Ni reacted with the substrate	[40]
Ni	Half- Heusler	Electro-brush Plating	Room Temperature	5.5	Good adhesion, low sheet resistance, high thermal stability, low cost/flat substrate surface requirement	This Study

As an alternative, the low-cost electro-brush plating technique offers high-quality coating deposition on conductive substrates [41]. In addition, this technique offers several advantages over the above-mentioned coating or metallisation methods. For instance, it enables precise thickness control, allowing selective surface metallisation without the need for masking or vacuum equipment [42]. This process also operates at low temperatures, thereby minimising thermal stress and preserving the structural integrity of the underlying thermoelectric material. While magnetron sputtering is a common technique for depositing metallic layers, it poses challenges for Ni due to its strong magnetic properties, which can distort the plasma and lead to non-uniform deposition. In contrast, brush plating avoids such issues, providing a uniform and adherent Ni layer [43]. From a device performance perspective, brush plating can produce coatings with high electrical conductivity and excellent interfacial adhesion, which are critical for reducing contact resistance and enhancing the overall efficiency, durability, and reliability of thermoelectric modules.

Therefore, in this study, pure and adherent Ni coatings were deposited on SS and P-type (Zr,Ti)Co(Sn,Sb) HH material to achieve a stable, highly conductive contact using the low-cost electro-brush plating method. The plating process was optimised on the SS substrate, and the surface morphology, microstructure, and mechanical properties of the Ni coating layers were investigated. The optimised parameters were subsequently used to deposit a Ni coating on the P-type (Zr,Ti)Co(Sn,Sb) TE material. In addition to the comprehensive characterisation of the Ni layer, its thermal stability on the TE material was evaluated at 500 °C for 10 h. It should be noted that this work focuses exclusively on single-layer Ni coatings and does not address other metallisation systems (e.g., Ag, Cu, or multilayer contacts) or module-level performance evaluation. Overall, this study provides a promising strategy for achieving a desired contact not only for thermoelectric materials but also for other conductive materials that require low sheet resistance at their joints.

## 2. Materials and Methods

### 2.1. Materials

316 stainless steel (SS, RS Components, Corby, UK) was used to optimise the Ni coating process, and its chemical composition is given in Table 2. A commercially available SS bar with a diameter of 25.4 mm was cut into five mm-thick discs. The samples were ground with silicon carbide (SiC) abrasive paper up to 1200 grit, then cleaned in an ultrasonic bath using deionised water, followed by acetone, each for 10 min, before the coating process.

Subsequently, the optimised Ni coating was deposited on the P-type (Zr,Ti)Co(Sn,Sb) HH TE material (MBN Nanomaterialia Scrl, Treviso, Italy) as a metallising contact layer. The TE material was produced through high-energy ball milling of commercially obtained Zr, Ti, Co, Sn and Sb powders in an inert environment (Ar) and sintering at 800 °C for 4 min under a pressure of 500 kg/cm^2^ using a uniaxial press (DSP 475, Dr. Fritsch, Fellbach, Germany). The chemical composition of the produced material is given in Table 3. The same sample preparation procedure used for the SS material was followed to prepare the P-type material before electro-brush plating Ni layers.

### 2.2. Electro-Brush Plating Ni Coating Process

Ni coatings were deposited on the SS and HH materials using an electro-brush plating kit (SPA Plating, Bath, UK), controlled by DC voltage. The plating kit includes an anode integrated with a pen holding the carbon electrode, and a cathode with a crocodile clip in contact with the sample. The electrode covered by cotton was periodically immersed in the commercial Ni ductile solution (supplied by SPA Plating) every ten seconds to prevent drying. In the first stage of the plating process, the samples’ surfaces were cleaned with a commercial electro-cleaning solution at 10 V for 1 min. Then, a commercial Ni ductile solution (supplied by SPA Plating) was used to plate Ni on the SS material at voltages ranging from 4 V to 8 V for 30 min. An optimum voltage of 6 V was determined for Ni deposition on the SS material and subsequently applied to the TE material for 10 min, given that the TE material’s electrical conductivity is higher than that of the SS material. The processing parameters and the sample codes are summarised in Table 4.

### 2.3. Thermal Stability Testing

The thermal stability of the Ni layer on the P-type TE material was evaluated by progressive oxidation at 500 °C for 10 h, and the results were compared with those of the oxidised uncoated material to assess the coating’s durability at moderate temperatures and the effectiveness of Ni in providing oxidation protection. It should be noted that the oxidation behaviour of (Zr,Ti)Co(Sn,Sb) TE material was comprehensively investigated in our previous studies [44,45]. The samples were placed in a ceramic container to allow precise handling during loading and unloading from a muffle furnace (Pyrotherm Furnaces, Leicestershire, UK). To minimise the risk of thermal shock, the samples were inserted into and removed from the furnace at a controlled temperature of 350 °C.

### 2.4. General Characterisation

The microstructure, surface morphology, and cross-section of the Ni coating samples were analysed using a scanning electron microscope (SEM, Jeol UK Ltd., Welwyn Garden City, UK), with an accelerating voltage of 20 kV and a working distance of 10 mm.

Elemental mapping and composition analysis were performed using an energy-dispersive spectroscopy (EDX, Oxford Instruments, High Wycombe, UK) under identical imaging conditions, with an acquisition time of 60 s per map and a count rate of 20–25 kcps. All SEM and EDX measurements were conducted at room temperature, and the data were processed using AZtec software (V5.0) for elemental quantification. Phase constitutions were identified by a ProtoA XRD device with a Cu-Kɑ source (λ = 1.540598 Å), fixed resolution of 0.01493, step size of 0.5° (2θ), and dwell time of 2 s. The surface roughness was measured using an Ambios XP-200 profilometer (CN Tech, Wisbech, UK) at a scanning speed of 0.05 mm/s over an 8 mm length. Glow discharge optical emission spectroscopy (GDOES; Spectruma Analytik GmbH, Hof, Germany) was used to profile mass concentration with depth and to measure coating thickness. The analyses were carried out under an Ar atmosphere at 335 Pa over a 4 mm diameter.

The hardness of the Ni coating layers was measured via Vickers micro-indentation (Zwick-Roell, Ulm, Germany). The load increased gradually to 10 g in 10 s, and the holding time was 10 s. Hardness measurements were repeated at least five times for each sample, and the resulting average was calculated. In addition, scratch testing was performed on the N6 sample to assess the mechanical integrity of the Ni layer and its adhesion to the stainless steel. Scratch testing was conducted using an ST30 Scratch Tester with a Rockwell spherical cone tip (Teer Coatings Ltd., Droitwich, UK), increasing the normal load from 5 N to 60 N at a constant speed of 10 mm/min across the coated surface. Additionally, the sheet resistance and electrical conductivity of the Ni coating layers were measured using a four-probe instrument (Ossila, Sheffield, UK) and compared to that of P-type TE material. During measurement, a constant outer current of approximately 2.5 × 10^−5^ A was applied, and the corresponding inner voltage drop of around 1.3 × 10^−3^ V was recorded. The probe spacing was 1 mm, and each value was averaged over at least five different surface points to minimise local variation.

## 3. Results

### 3.1. Ni Coatings on Stainless Steel

#### 3.1.1. Depth Profiling

**Figure 1:**
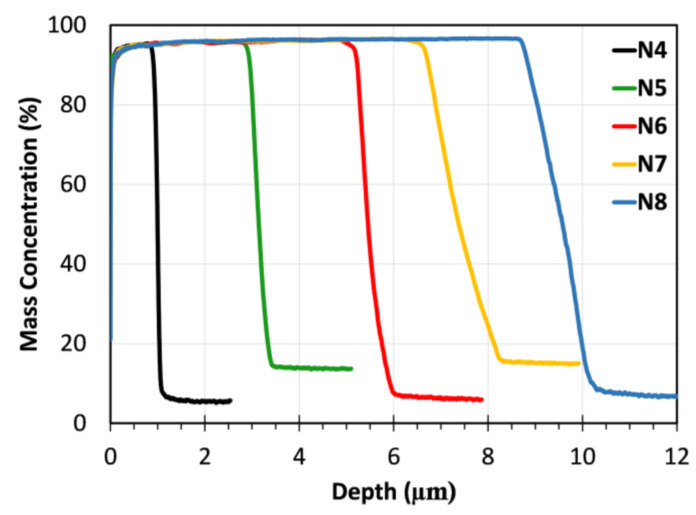
Mass concentration of the Ni coatings along the depth.

Depth profile analyses of Ni-coated SS samples at different voltages (4 to 8 V) are shown in Figure 1. As expected, increasing the applied voltage increased coating thickness due to the stronger electric field, which accelerated ion transport, leading to thicker coatings in a short time. The thinnest Ni coating was observed for the N4 sample (Ni coating on SS sample using 4 V over 30 min) with a thickness of approximately 1.5 μm. With increasing voltage, the coating thickness increased progressively to ~3.2 μm for N5 (Ni coating on SS sample using 5 V over 30 min), ~5.5 μm for N6 (Ni coating on SS sample using 6 V over 30 min), ~7.7 μm for N7 (Ni coating on SS sample using 7 V over 30 min), and reached its maximum value of ~10.5 μm for N8 (Ni coating on SS sample using 8 V over 30 min). Notably, the Ni concentration remains close to 100% throughout the coating thickness in all samples, indicating uniform deposition. The sharp drop at the interface indicates minimal interdiffusion between the Ni layer and the SS.

#### 3.1.2. Coatings Microstructure

**Figure 2:**
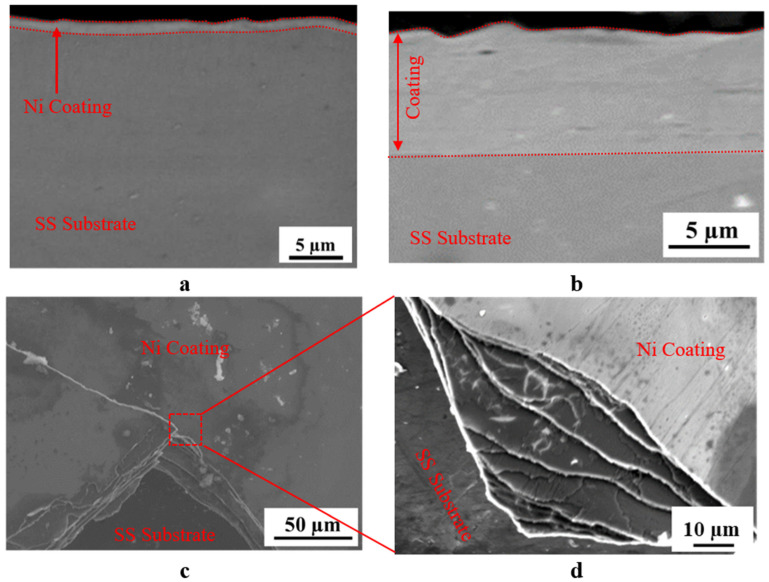
SEM images of the produced coatings: (**a**) Cross section of N4 sample, (**b**) cross section of N6 sample, (**c**) crack formation during plating in the N8 sample, (**d**) higher magnification of the fractured coating layer of the N8 sample showing the layer-by-layer structure.

The cross-sectional SEM images of the N4 (Figure 2a) and N6 samples reveal a uniform Ni coating with good adhesion to the stainless steel, indicating high-quality deposition (Figure 2b). Although the coating thickness varied with voltage, the coating quality (adhesion and no degradation) of N5 is similar to that of N4 and N6. On the other hand, for the N7 and N8 samples, the coating at the edges was degraded during brushing, indicating that coating quality decreased at high voltages, as shown in Figure 2c,d. The fractured coating layer was further investigated by SEM at high magnification (Figure 2d), revealing that the Ni coatings grew in a layer-by-layer structure. As is known, the carbon electrode wrapped in cotton is dipped into the plating solution every 10 s. It carries the plating on, meaning the process is a 10-s deposition repeated over 30 min. With each brushing, a nanosized coating layer is deposited on the sample surface, forming a multilayered structure as it accumulates and continues to grow as plating proceeds.

#### 3.1.3. Phase Constitution Change

**Figure 3:**
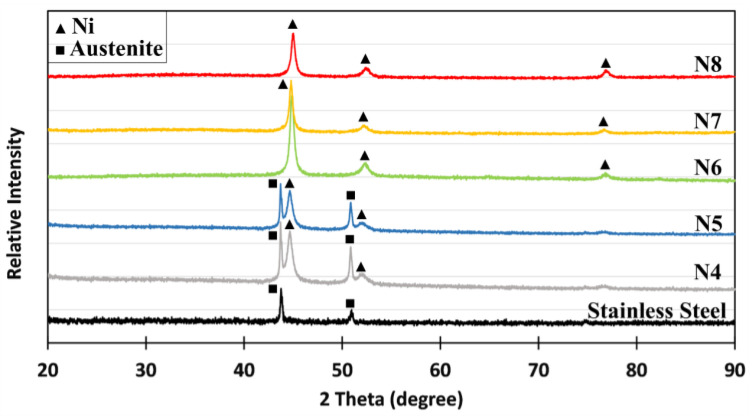
Typical XRD patterns of Ni coatings.

XRD patterns of the Ni coatings deposited at lower plating voltages (samples N4 and N5 in Figure 3), where the coating thickness was less than 3 µm, exhibited crystallographic reflections corresponding to the face-centred cubic (FCC) Ni phase. Additionally, peaks associated with the austenitic SS were observed, indicating that X-rays penetrated the SS via the Ni layer. In contrast, no austenite-related peaks were detected for coatings deposited at higher voltages (samples N6, N7, and N8), suggesting that the thicker Ni layer prevented X-ray penetration to the stainless steel.

#### 3.1.4. Surface Roughness

**Figure 4:**
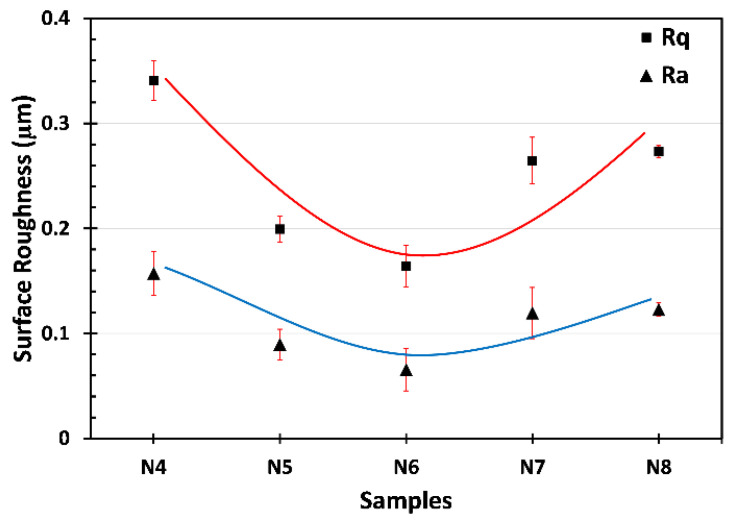
Surface roughness of the produced Ni coatings.

Average roughness (Ra) and root mean square roughness (Rq) were measured for each sample, and the results are given in Figure 4. The lowest surface roughness was observed for sample N6 with Ra ≈ 0.7 μm and Rq ≈ 0.17, whereas the highest surface roughness was measured for sample N4 with Ra ≈ 1.6 μm and Rq ≈ 0.34 μm. The lower deposition rate at lower voltages resulted in larger, irregular Ni grains, thereby increasing surface roughness. On the other hand, the adhesion of rapidly nucleating Ni particles to the SS does not occur at the desired quality at high deposition rates, which causes partial peeling and increases the surface roughness as seen in N7 and N8 samples in Figure 4. Additionally, high-rate deposition increases the internal stress within the coating, leading to degraded coating and a rougher surface.

#### 3.1.5. Mechanical Properties of the Ni Coatings

**Figure 5:**
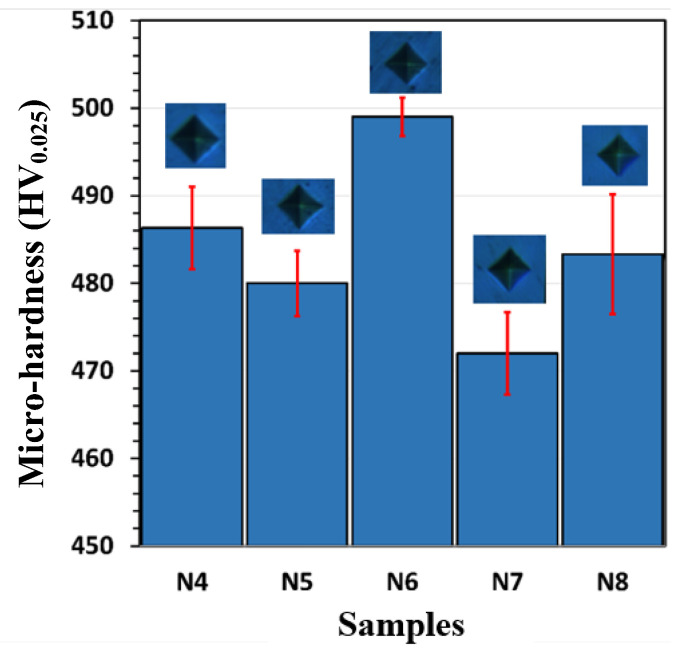
Microhardness of the produced Ni coatings.

The hardness for the Ni-coated samples (N4 to N8 in Figure 5) demonstrated significant variation with the applied voltage during electro-brush plating. Sample N6 exhibited the highest hardness at HV_0.025_ 498, with minimal variation. At the same time, the lowest value was obtained for the N7 sample at around HV_0.025_ 472, indicating that a plating voltage of 6 V is optimal for producing a layer with higher hardness, likely due to a fine-grain structure, and for improving the uniformity of the coating.

**Figure 6:**
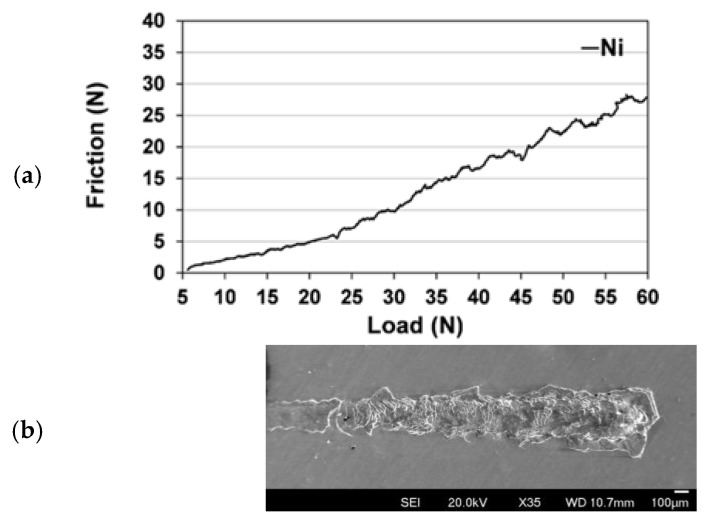
Scratch test on the N6 sample; (**a**) friction force against the load applied, (**b**) SEM image of the scratched surface.

The adhesion of the Ni coating on the N6 sample was evaluated through progressive load scratch testing, and the resulting friction force-load graph is given in Figure 6a. The frictional force exhibited a steady increase with the applied normal load, indicating a gradual response of the coating to increasing mechanical stress. Initially, at lower loads (~5 to 15 N), the friction force remained relatively low, suggesting a good interfacial stability between the coating and SS. However, as the load increased to 30 N, a noticeable rise in friction was observed, and local fluctuations became more pronounced, indicating the onset of micro-cracking or partial delamination. This is supported by the SEM image in Figure 6b, which shows extensive cracking, material displacement, and coating detachment along the scratch track on the N6 sample. The morphology of the scratch suggests that the Ni coating maintained good adhesion up to a critical load of 30 N.

#### 3.1.6. Sheet Resistance and Electrical Conductivity

**Figure 7:**
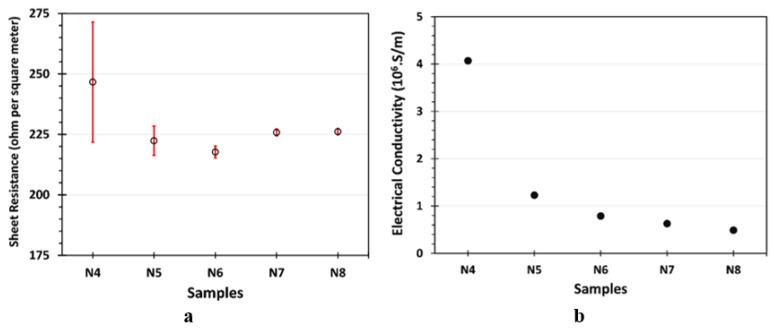
(**a**) Sheet resistance and (**b**) electrical conductivity of Ni layers.

The sheet resistances of the Ni-coating samples are shown in Figure 7a. As shown, N4 exhibited the highest sheet resistance of approximately 248 ohms per square meter, with significant variability, possibly due to the inconsistent thickness of the Ni layer. Similar results were obtained for the N5 sample, with a smaller error bar than for N4. The sheet resistance of the N6, N7, and N8 samples showed greater consistency, with values similar to one another (Table S1). It is known that thinner coatings generally have higher sheet resistance due to reduced conductive pathways and increased contributions from surface roughness, defects, and the substrate. The thicker N6, N7, and N8 coatings likely contributed to lower, more consistent sheet resistance values. It should be noted that the measurements were taken from the undegraded regions of the N7 and N8 samples.

The electrical conductivity values of the Ni layers are presented in Figure 7b. These values were calculated using the equation, σ = 1/(R_s_ × t), where R_s_ is the sheet resistance (in Ω/m^2^), and t is the coating thickness (in meters). The thicknesses of the Ni layers for each sample were obtained from the cross-sectional measurements, as shown in Figure 1, while the corresponding sheet resistance values were taken from Figure 7a. As shown in Figure 7b, the N4 sample exhibited the highest electrical conductivity, approximately 4.07 × 10^6^ S/m, due to its very thin Ni layer. However, its significant variability, as observed in the sheet resistance (Figure 7a), suggests poor uniformity and coverage at this thickness. The conductivity gradually decreased with increasing coating thickness from N5 to N8, reaching a value around 0.49 × 10^6^ (S/m) for the N8 sample. Although thicker films on the N6, N7, and N8 samples reduced overall conductivity, they provided more consistent and reliable performance, likely due to better coverage and reduced influence of substrate effects (Table S2).

### 3.2. Ni Coating on the P-Type (Zr,Ti)Co(Sn,Sb) TE Material

Following a comprehensive characterisation of Ni coatings deposited on SS, an optimum plating voltage of 6 V was identified and selected for the deposition of Ni coatings on TE material. At 6 V, a uniform, well-adhered Ni layer approximately six μm thick was achieved, as confirmed by SEM cross-sectional analysis. This coating exhibited the lowest surface roughness (Ra ~ 0.7 μm), highest hardness (~498 HV), and consistent electrical properties, indicating superior overall quality compared to coatings deposited at both lower and higher voltages. Lower voltages yielded thin, non-uniform layers with greater variability, whereas higher voltages led to coating edge degradation due to excessive internal stresses and poor adhesion. Therefore, 6 V was deemed the optimal condition and subsequently applied to deposit a Ni layer on the P-type (Zr,Ti)Co(Sn,Sb) half-Heusler thermoelectric material to ensure both electrical functionality and structural stability.

#### 3.2.1. Coating Structure

**Figure 8:**
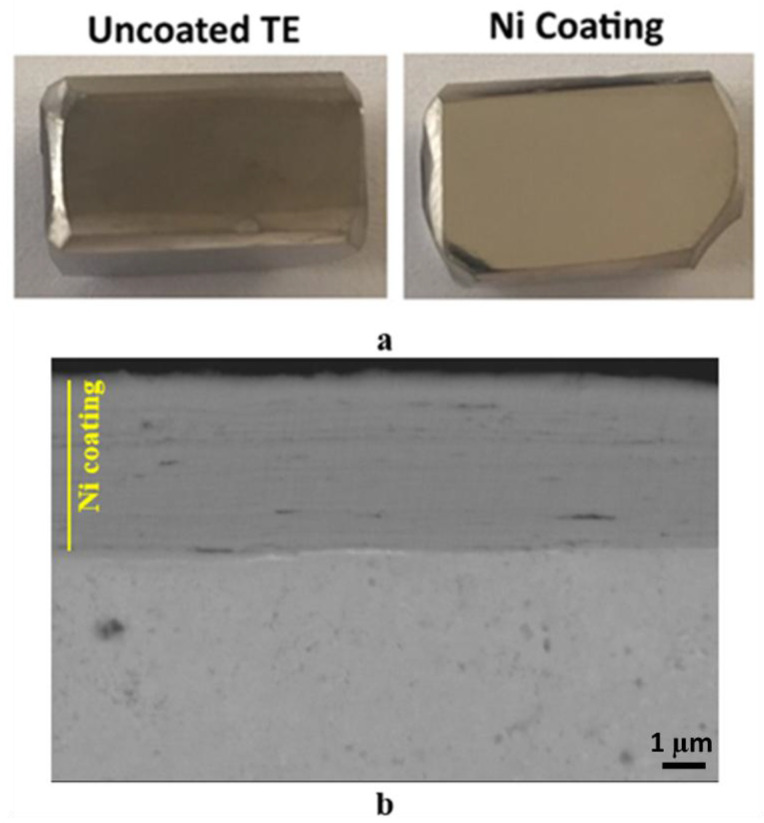
(**a**) Images of the deposited coatings on the P-type TE material compared with the uncoated sample, and (**b**) cross-sectional backscattered electron microscope image of the coating layer.

Macro images of the uncoated and Ni-coated P-type TE materials are presented in Figure 8a, comparing the samples’ surfaces before and after the coating process. It was observed that Ni was successfully deposited onto the P-type material, with no evidence of peeling or degradation during plating. The surface of the Ni-plated sample appears shiny, with a smooth, uniform coating. Cross-sectional analysis further confirms the presence of the Ni layer on the TE material (Figure 8b), with a thickness of approximately 6 µm. The coating displays a layer-by-layer structure, similar to that of Ni on the SS. This structural uniformity suggests effective deposition and strong adhesion, contributing to the coated material’s mechanical and functional integrity.

#### 3.2.2. Thermal Stability of Ni Coating on the P-Type (Zr,Ti)Co(Sn,Sb) TE Materials

**Figure 9:**
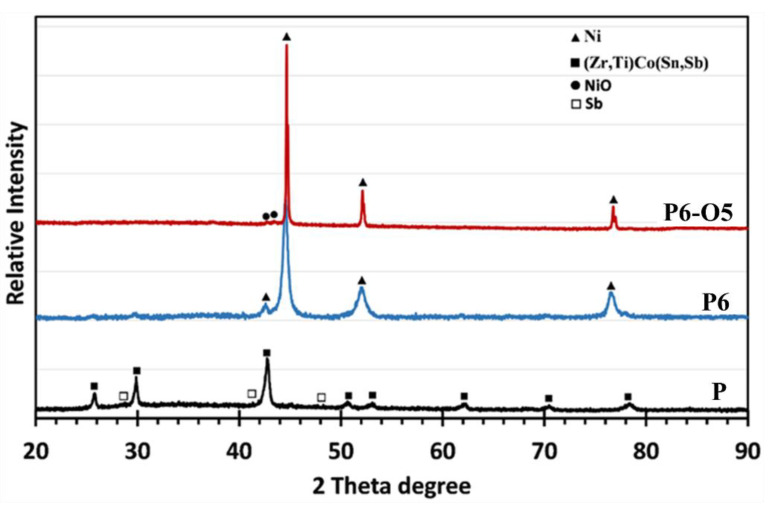
XRD patterns of P6 (Ni-coated P-type material) and P6-O5 (Ni-coated and oxidised P-type material) samples compared with the as-received P sample.

XRD patterns of as-received P-type TE material were compared with the Ni-plated samples before and after static oxidation testing, as displayed in Figure 9. Patterns from as-produced P-type material identified a half-Heusler phase of (Zr,Ti)Co(Sn,Sb) with firm peaks. In addition, weak traces of the Sb phase were detected. As expected, high-intensity Ni peaks were identified for the Ni-plated sample (P6). After oxidation, in addition to the Ni peaks observed for the P6 sample, very weak NiO peaks were identified for the P6-O5 sample, indicating the formation of a very thin surface oxide layer on the coating. It was also observed that the full width at half maximum (FWHM) of the Ni peaks decreased considerably after oxidation, indicating that the Ni grain size on the sample increased with oxidation. Notably, no oxide peaks from the TE material were observed in the Ni-coated material, indicating that the coating protected it against oxidation.

**Figure 10:**
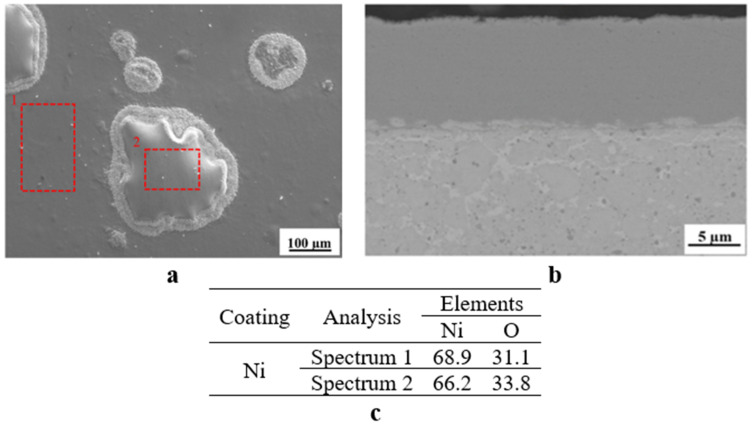
(**a**) Surface morphology, (**b**) cross-section of the Ni coating samples after oxidation testing, and (**c**) EDX analysing results of selected regions.

The SEM and EDX analyses further characterised the surface of the P6-O5 sample, and the obtained images and data are presented in Figure 10. As shown in Figure 10a, some part of the Ni coating was bubbled and several small-sized oxide particles were formed around these bubble-like growths, which are also rich in oxygen (see Figure 10c). However, the cross-sectional SEM image of the P6-O5 sample shows that bubble-like growths are formed just on the very top surface of the coating (Figure 10b), meaning that the majority of the Ni coating is still in the original form on the TE material. It is also seen that the oxygen quantity on the surface of the coating is higher than that at the interface (Figure 10c), which is in line with the XRD results given in Figure 9.

**Figure 11:**
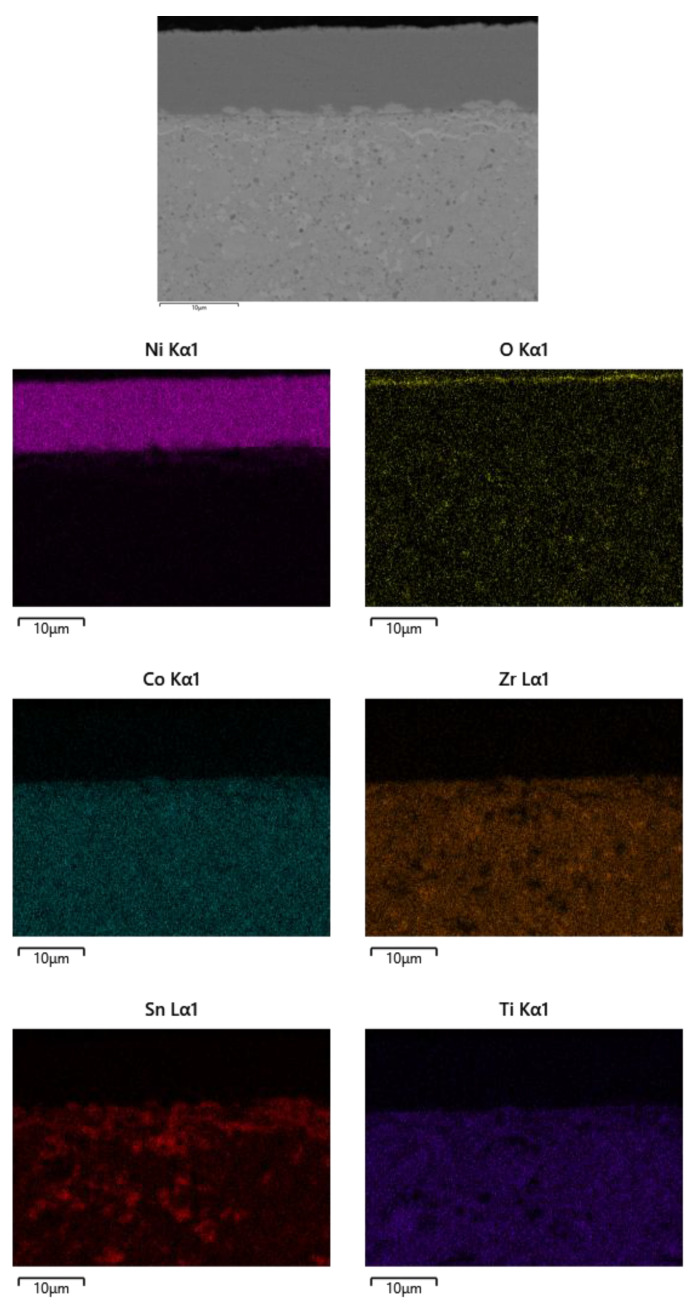
Elements mapping of the cross-section view of the oxidised Ni coating.

The element distribution was investigated across the cross-section of the P6-O5 sample by EDX elemental mapping, and the obtained results are presented in Figure 11. The oxygen concentration at the top of the coating layer is very high, meaning that a superficial nickel oxide layer would form on the top surface of the coating, as confirmed by the XRD results (Figure 9), which blocks oxygen inward diffusion into the Ni coating and the TE material, thus preventing oxidation of the TE material. Notably, some ignorable elemental diffusion at the interface between the coating and substrate material was observed. As previously mentioned, TE material has Sn and Sb, which can sublimate at moderate and high temperatures. Fortunately, the Ni coating significantly prevented sublimation of those elements and provided microstructural stability at 500 °C for 10 h.

## 4. Discussion

Among the produced Ni coatings, the N6 sample exhibited superior overall performance in terms of its electrical, mechanical, and microstructural properties. As is well known, electrical conductivity plays a crucial role in determining the suitability of contact layers for thermoelectric materials and modules. Based on the measurements presented in Figure 7a, N6 showed a sheet resistance of 217 Ω/m^2^, which, combined with its coating thickness of 5.8 µm (see Figure 1), resulted in a calculated electrical conductivity of approximately 8.5 × 10^6^ S/m. This value is significantly higher than that of the uncoated bulk P-type TE material (~2.3 × 10^5^ S/m), clearly indicating an enhancement in electrical conductivity. As is known, sheet resistance is a critical parameter for evaluating the electrical performance of contact layers in thermoelectric devices. It reflects the ease with which charge carriers can move laterally through the coating and, consequently, determines the interfacial and contact resistances at junctions. A lower sheet resistance indicates a more conductive surface, reducing parasitic ohmic losses and enhancing current flow between the thermoelectric leg and the electrode. Therefore, this improvement (reduction) in the sheet resistance of the N6 sample is insightful for ensuring effective charge transport and a high power factor in a TE module. Moreover, N6 coating achieved this thickness with minimal surface roughness (Figure 4), without the degradation observed in N7 and N8 samples (see Figure 2b,c), thereby forming a well-connected metallic pathway.

From a mechanical property perspective, the N6 sample was harder than the other samples, further supporting the benefit of medium-speed Ni deposition at 6 V. In contrast, lower deposition voltages (4 V and 5 V) generally resulted in slower growth rates and thinner coatings (see Figure 1), which can lead to incomplete substrate coverage or less compact microstructures. These microstructural limitations can significantly reduce the coating’s resistance to mechanical stress. In addition, excessively high deposition rates led to poor layer adhesion, resulting in degradation during the coating process. The scratch test further confirmed the optimal mechanical performance of the N6 sample (Figure 6a), which revealed a stable and gradually increasing friction response under increasing load, indicating sufficient adhesion of the Ni layer to the SS.

It was also essential to evaluate the thermal stability of the optimised Ni layer, produced under the optimum plating parameters, on TE material at 500 °C, which represents one of the real-life operation conditions of HH materials. Although superficial, bubble-like defects were observed on the surface of the P6 sample, the Ni layer served as an effective barrier against oxidation, sublimation, and interfacial degradation between the TE materials and the electrodes. In summary, the Ni layer deposited via the low-cost electro-brush plating technique strategically enhanced the interfacial contact properties of P-type HH material.

## 5. Conclusions

In this study, the Ni metallising coatings were characterised and optimised on the SS and subsequently applied to P-type (Zr,Ti)Co(Sn,Sb) thermoelectric material using a low-cost electro-brush plating technique. The experimental findings can be concluded as follows:

- The electro-brush plating technique successfully deposited the Ni coating on both SS and P-type thermoelectric material. An applied voltage (6 V), providing a medium-rate deposition, was critical for achieving a high-quality layer without degradation or peeling during the plating.
- The plated Ni coating has conductivity higher than that of the bulk TE material, which is beneficial in improving charge transport at the interface.
- The microstructure of the Ni coating deposited on the P-type material was consistent with that observed on the SS. The coating adhered well to the TE material, forming a dense and uniform multilayered structure.
- The Ni layer provided microstructural stability to the TE materials after oxidation, highlighting its protective role against oxidation and sublimation at 500 °C. The formation of a very thin nickel oxide layer on the coating surface effectively suppressed oxygen diffusion, thereby preventing further oxidation.

In short, the interfacial contact properties of P-type (Zr,Ti)Co(Sn,Sb) TE material can be effectively enhanced by depositing a highly conductive, oxidation-resistant Ni layer using the cost-effective, straightforward electro-brush plating technique. Future studies will extend this coating approach to other thermoelectric materials, including Mg_2_Si-based and various half-Heusler systems, to assess their long-term thermal stability and interfacial performance and to enable reliable and effective TE module performance at elevated temperatures.

## Figures and Tables

**Table 2:** Chemical composition (at.%) of AISI 316 SS.

	C	Si	Mn	Cr	Ni	Mo	Fe	Other
AISI 316	0.03	1.00	2.00	16.50–18.50	10.00–13.00	2.00–2.50	Bal.	<1.00

**Table 3:** Chemical composition (at.%) of the P-type HH material.

	Zr	Ti	Co	Sn	Sb
(Zr,Ti)Co(Sn,Sb)	18.1	16.4	29.1	10.7	25.7

**Table 4:** Sample codes and their corresponding plating parameters.

Sample Codes	Substrate	Voltage (V)	Deposition Time (min)	Oxidation Test
N4	SS	4	30	-
N5		5		
N6		6		
N7		7		
N8		8		
P6	(Zr,Ti)Co(Sn,Sb)	6	10	-
P6-O5				500 °C/10 h

## Data Availability

The original contributions presented in this study are included in the article. Further inquiries can be directed to the corresponding authors.

## Supplementary Materials

The following supporting information can be downloaded at: https://www.mdpi.com/article/10.3390/ma18225108/s1, Table S1: Sheet resistance of the produced Nİ coatings on the SS substrate; Table S2: The calculated electrical conductivity of Ni coatings on the SS substrate as a function of sheet resistance and coating thickness.

## Abbreviations

The following abbreviations are used in this manuscript:

DC	Direct current
EDX	Energy-dispersive X-ray spectroscopy
FCC	Face-centred cubic
FWHM	Full width at half maximum
GDOES	Glow discharge optical emission spectroscopy
HH	Half Heusler
min.	Minute(s)
SEM	Scanning electron microscopy
SiC	Silicon carbide
SS	Stainless steel
TE	Thermoelectric
V	Volt
XRD	X-ray diffraction
zT	Figure of merit

## References

[1] Ganechari S., Kate S. (2005). Alternative Energy Sources.

[2] Bobokulova M. (2024). Alternative energy sources and their use. Med. Pedagog. Technol. Theory Pract..

[3] Liu W., Hu J., Zhang S., Deng M., Han C.-G., Liu Y. (2017). New trends, strategies and opportunities in thermoelectric materials: A perspective. Mater. Today Phys..

[4] Gayner C., Kar K.K. (2016). Recent advances in thermoelectric materials. Prog. Mater. Sci..

[5] Ma Z., Wei J., Song P., Zhang M., Yang L., Ma J., Liu W., Yang F., Wang X. (2021). Review of experimental approaches for improving zT of thermoelectric materials. Mater. Sci. Semicond. Process..

[6] Wei J., Yang L., Ma Z., Song P., Zhang M., Ma J., Yang F., Wang X. (2020). Review of current high-ZT thermoelectric materials. J. Mater. Sci..

[7] Han H., Zhao L., Wu X., Zuo B., Bian S., Li T., Liu X., Jiang Y., Chen C., Bi J. (2024). Advancements in thermoelectric materials: Optimization strategies for enhancing energy conversion. J. Mater. Chem. A.

[8] Moshwan R., Shi X.-L., Liu W.-D., Liu J., Chen Z.-G. (2024). Entropy engineering: An innovative strategy for designing high-performance thermoelectric materials and devices. Nano Today.

[9] Kumar A., Bano S., Govind B., Bhardwaj A., Bhatt K., Misra D. (2021). A review on fundamentals, design and optimization to high ZT of thermoelectric materials for application to thermoelectric technology. J. Electron. Mater..

[10] Snyder G.J., Snyder A.H. (2017). Figure of merit ZT of a thermoelectric device defined from materials properties. Energy Environ. Sci..

[11] He Q., Yang D., Xia S., Song H. (2024). Ultra-low thermal conductivity and improved thermoelectric performance in La_2_O_3_-dispersed Bi_2_Sr_2_Co_2_O_y_ ceramics. Mater. Sci. Eng. B.

[12] Lim K.H., Li M., Zhang Y., Wu Y., Zhou Q., Wang Q., Yang X., Liu P., Wang W.-J., Wong K.W. (2024). Modulation doping of *P*-type Cu12Sb4S13 toward improving thermoelectric performance. J. Mater. Sci. Technol..

[13] Yu J., Xing Y., Hu C., Huang Z., Qiu Q., Wang C., Xia K., Wang Z., Bai S., Zhao X. (2020). Half-Heusler thermoelectric module with high conversion efficiency and high power density. Adv. Energy Mater..

[14] Schierning G., Chavez R., Schmechel R., Balke B., Rogl G., Rogl P. (2015). Concepts for medium-high to high temperature thermoelectric heat-to-electricity conversion: A review of selected materials and basic considerations of module design. Transl. Mater. Res..

[15] Rebellon H.E., Henao O.F.P., Gutierrez-Velasquez E.I., Amell A.A., Colorado H.A. (2024). Thermoelectric modules: Applications and opportunities in building environments for sustainable energy generation: From biomass, municipal waste, and other sources. Eng. Sci..

[16] Sun W., Sui R., Yuan G., Zheng H., Zeng Z., Xie P., Yuan L., Ren Z., Cai F., Zhang Q. (2021). Thermoelectric module design to improve lifetime and output power density. Mater. Today Phys..

[17] Zhao J., Kuang Z., Long R., Liu Z., Liu W. (2024). Impacts of thermal and electric contact resistance on the material design in segmented thermoelectric generators. Energy Storage Sav..

[18] Tian Y., Ren G.-K., Wei Z., Zheng Z., Deng S., Ma L., Li Y., Zhou Z., Chen X., Shi Y. (2024). Advances of thermoelectric power generation for room temperature: Applications, devices, materials and beyond. Renew. Energy.

[19] Wederni A., Daza J., Ben Mbarek W., Saurina J., Escoda L., Suñol J.-J. (2024). Crystal structure and properties of Heusler alloys: A comprehensive review. Metals.

[20] Soltanbek N.S., Merali N., Sagatov N.E., Abuova F.U., Elsts E., Abuova A.U., Khovaylo V., Inerbaev T., Konuhova M., Popov A.I. (2025). Ab initio investigation of the stability, electronic, mechanical, and transport properties of a new double Half-Heusler alloys Ti2Pt2ZSb (Z = Al, Ga, In). Metals.

[21] Kawasaki J.K., Chatterjee S., Canfield P.C., Editors G. (2022). Full and half-Heusler compounds. MRS Bull..

[22] Gurunani B., Ghosh S., Gupta D.C. (2024). Comprehensive investigation of half Heusler alloy: Unveiling structural, electronic, magnetic, mechanical, thermodynamic, and transport properties. Intermetallics.

[23] Li W., Ghosh S., Liu N., Poudel B. (2024). Half-Heusler thermoelectrics: Advances from materials fundamental to device engineering. Joule.

[24] Chen R., Kang H., Min R., Chen Z., Guo E., Yang X., Wang T. (2024). Thermoelectric properties of half-Heusler alloys. Int. Mater. Rev..

[25] Le W., Yang W., Sheng W., Shuai J. (2023). Research progress of interfacial design between thermoelectric materials and electrode materials. ACS Appl. Mater. Interfaces.

[26] Li Y., Shi Y., Luo D., Wang X., Yan Y. (2024). Impacts of distributed thermal and electric contact resistance on performance and geometric optimization of thermoelectric generators. Appl. Therm. Eng..

[27] Shtern Y., Mironov R., Shtern M., Sherchenkov A., Rogachev M. (2016). Technology and investigation of ohmic contacts to thermoelectric materials. Acta Phys. Pol. A.

[28] Zhang Z., Gurtaran M., Dong H. (2024). Low-Cost Magnesium-Based Thermoelectric Materials: Progress, Challenges, and Enhancements. ACS Appl. Energy Mater..

[29] Korchagin E., Shtern M., Petukhov I., Shtern Y., Rogachev M., Kozlov A., Mustafoev B. (2022). Contacts to thermoelectric materials obtained by chemical and electrochemical deposition of Ni and Co. J. Electron. Mater..

[30] Korchagin E., Shtern M.Y., Petukhov I., Shtern Y.I., Rogachev M., Kozlov A., Mustafoev B., Dedkova A. (2022). Formation and Properties of Nickel Contacts to Thermoelectric Materials Based on Bismuth and Antimony Chalcogenides. Russ. J. Appl. Chem..

[31] Ayachi S., Castillo Hernandez G., Pham N.H., Farahi N., Müller E., de Boor J. (2019). Developing Contacting Solutions for Mg2Si1–x Sn x-Based Thermoelectric Generators: Cu and Ni45Cu55 as Potential Contacting Electrodes. ACS Appl. Mater. Interfaces.

[32] Zhang H., Wei P., Zhou C., Li L., Nie X., Zhu W., Zhao W. (2024). Improved contact performance and thermal stability of Co–Ni alloy barrier layer for bismuth telluride-based thermoelectric devices. J. Mater. Sci. Mater. Electron..

[33] Basu R. (2023). Thermoelectric modules: Key issues in architectural design and contact optimization. ChemNanoMat.

[34] Korchagin E., Shtern Y.I., Petukhov I., Shtern M.Y., Rogachev M., Sherchenkov A., Kozlov A., Ryazanov R. (2024). Chemical Solution Deposition of Nickel and Cobalt Contacts on Catalytically Active Surfaces of Thermoelectric Materials. Inorg. Mater..

[35] Kumari N., Dasgupta T. (2023). Developing contacting solutions for n-type Mg_3_Sb_1.5_Bi_0.5_ based thermoelectric materials. J. Alloys Compd..

[36] Fang C., Chen G., Li Z., Wang X., Duan B., Feng X., Li G., Zhai P. (2025). Mechanical bonding and metallurgical bonding synergistically optimize the shear properties of the Ni/Bi_2_Te_3_ joint interface. J. Mater. Sci. Mater. Electron..

[37] Tan M., Liu W.-D., Shi X.-L., Sun Q., Chen Z.-G. (2023). Minimization of the electrical contact resistance in thin-film thermoelectric device. Appl. Phys. Rev..

[38] Ioannou P., Mangelis P., Hadjipanteli S., Mina M., Søiland A., Giapintzakis J., Kyratsi T. (2025). Development of silicide thermoelectric modules based on high purity Si and recycled Si-kerf: A contact resistance investigation. Mater. Today Energy.

[39] Shtern M.Y., Sherchenkov A., Shtern Y.I., Rogachev M., Korchagin E. (2023). Preparation of the Thermoelement Surfaces and Investigation of Ohmic Film Contacts Formed on Them by Different Methods. J. Surf. Investig. X-Ray Synchrotron Neutron Tech..

[40] Zhang J., Wei P., Zhang H., Li L., Zhu W., Nie X., Zhao W., Zhang Q. (2023). Enhanced contact performance and thermal tolerance of Ni/Bi_2_Te_3_ joints for Bi_2_Te_3_-based thermoelectric devices. ACS Appl. Mater. Interfaces.

[41] Chen X., Jiang N., Zhan Y., Luo Y., Ye N., Tang J., Zhuo H. (2024). Preparation and tribological performance of Ni-SiC composite coating on 304 stainless steel through brush plating. Surf. Coat. Technol..

[42] Wang W., Liu X., Shi Z. (2016). Mechanisms and influences of electro-brush plating micro-force on coatings performances. J. Mater. Res..

[43] Qi S., Li X., Dong H. (2019). Reduced friction and wear of electro-brush plated nickel composite coatings reinforced by graphene oxide. Wear.

[44] Gurtaran M., Zhang Z., Li X., Dong H. (2024). Cyclic oxidation behaviour of N-type (Zr, Ti) Ni (Sn, Sb) and P-type (Zr, Ti) Co (Sn, Sb) thermoelectric materials. J. Mater. Res. Technol..

[45] Gurtaran M., Zhang Z., Li X., Dong H. (2024). Developing advanced CrSi coatings for combatting surface degradation of N-type (Zr, Ti) Ni (Sn, Sb) and P-type (Zr, Ti) Co (Sn, Sb) half Heusler thermoelectric materials. J. Mater. Res. Technol..

