# Supplementary material for: Characterisation of Electro-Brush Plated Nickel Coatings on P-Type (Zr,Ti)Co(Sn,Sb) Half-Heusler Thermoelectric Materials for Stable Contact Layers
Source: Materials (Basel). 2025 Nov 10;18(22):5108. doi: 10.3390/ma18225108 (PMC12654000; doi:10.3390/ma18225108)
Supplement: Supplementary file 1 [file materials-18-05108-s001.zip › materials-3921192-supplementary.pdf]

# Supplementary Information

Table S1. Sheet resistance of the produced Nİ coatings on the SS substrate.

| Measurements | Sheet Resistance (ohm per square meter) | | | | |
|---|---|---|---|---|---|
| | **N4** | **N5** | **N6** | **N7** | **N8** |
| **1** | 191.25 | 236 | 215.78 | 224.43 | 221.77 |
| **2** | 182.16 | 230 | 223.87 | 221.21 | 226.29 |
| **3** | 292.15 | 225.11 | 222.6 | 227.52 | 225.48 |
| **4** | 271.85 | 220 | 215.63 | 226.45 | 228.11 |
| **5** | 295.68 | 200.73 | 210.72 | 229.32 | 228.88 |
| **Average** | 246.618 | 222.368 | 217.72 | 225.786 | 226.106 |
| **Deviation** | 24.83681 | 6.022491 | 2.436889 | 1.391318 | 1.243739 |

Table S2. The calculated electrical conductivity of Ni coatings on the SS substrate as a function of sheet resistance and coating thickness.

| Sample | Sheet Resistance (ohm per square meter) | Coating Thickness | Electrical Conductivity ($\times 10^6$ S/m) |
|---|---|---|---|
| N4 | 246.618 | 1.5 | 4.07 |
| N5 | 222.368 | 3.2 | 1.23 |
| N6 | 217.72 | 5.5 | 0.79 |
| N7 | 225.786 | 7.7 | 0.63 |
| N8 | 226.106 | 10.5 | 0.49 |
